# The impact of sound stimulations during pregnancy on fetal learning: a systematic review

**DOI:** 10.1186/s12887-023-03990-7

**Published:** 2023-04-20

**Authors:** Kobra Movalled, Anis Sani, Leila Nikniaz, Morteza Ghojazadeh

**Affiliations:** 1 Tabriz University of Medical Sciences, Tabriz, Iran; 2 Tabriz Health Services Management Research Center, Health Management and Safety Promotion Research Institute, Tabriz University of Medical Sciences, Tabriz, Iran; 3 Professor of Physiology, Iranian Centre for Evidence-Based Medicine, Tabriz University of Medical Sciences, Tabriz, Iran

**Keywords:** Sound stimulation, Pregnancy, Fetal learning, Neonatal behavior, Fetal memory, Neural development

## Abstract

**Background:**

The developing nervous system in utero is exposed to various stimuli with effects that may be carried forward to the neonatal period. This study aims to investigate the effects of sound stimulation (music and speech) on fetal memory and learning, which was assessed later in neonatal period.

**Methods:**

The MEDLINE (pubmed), Scopus, EMBASE, and Cochrane Library were searched. Two reviewers selected the studies and extracted the data independently. The quality of eligible studies was assessed using The Joanna Briggs Institute Critical Appraisal Checklist for Randomized Controlled Trials (RCTs).

**Results:**

Overall 3930 articles were retrieved and eight studies met the inclusion criteria. All of the included studies had good general quality; however, high risk of selection and detection bias was detected in most of them. Fetal learning was examined through neonatal electrocardiography (ECG), electroencephalography (EEG), habituation tests, and behavioral responses. Seven studies showed that the infants had learned the fetal sound stimulus and one study indicated that the prenatally stimulated infants performed significantly better on a neonatal behavior test. There was considerable diversity among studies in terms of sound stimulation type, characteristics (intensity and frequency), and duration, as well as outcome assessment methods.

**Conclusions:**

Prenatal sound stimulation including music and speech can form stimulus-specific memory traces during fetal period and effect neonatal neural system. Further studies with precisely designed methodologies that follow safety recommendations, are needed.

**Graphical Abstract:**

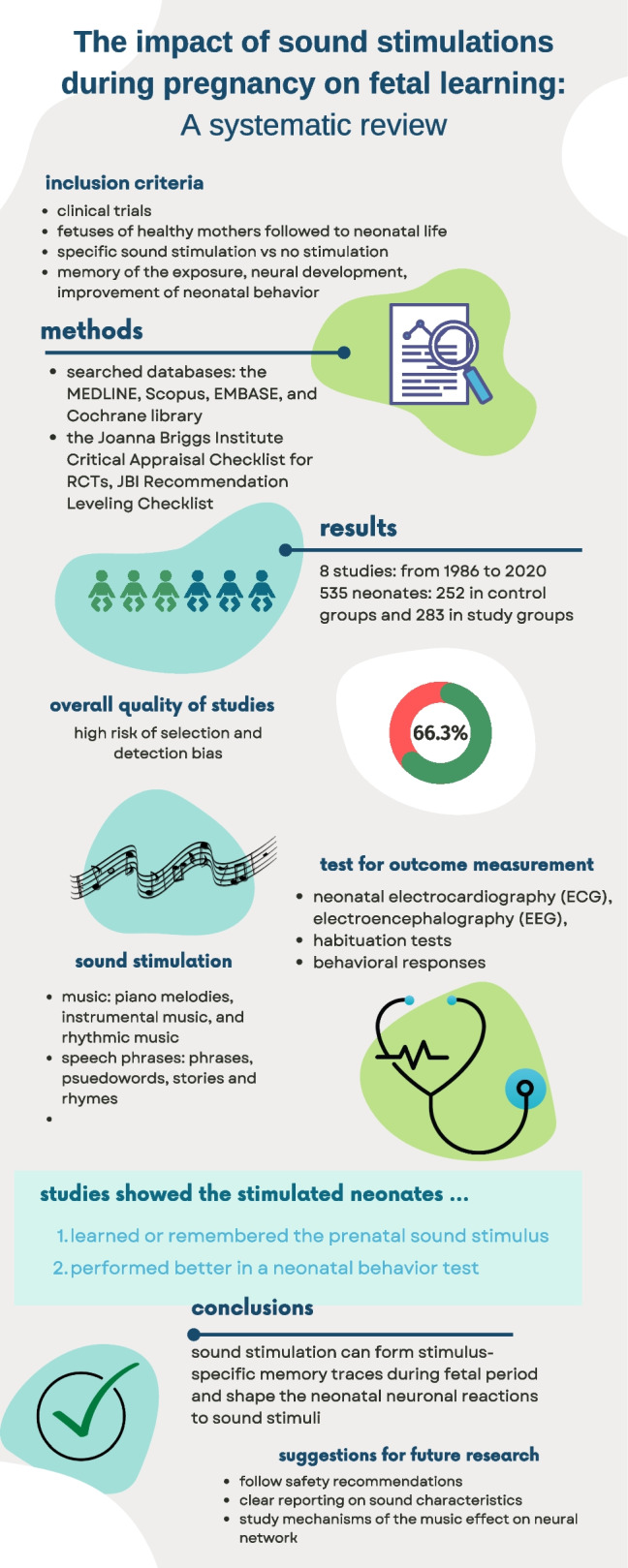

## Introduction

The developing nervous system in utero is exposed to various stimuli with effects that may be carried forward to the newborn period. The human brain is influenced by music experience, beginning in utero and lasting across the lifetime [1]. The Neural processing of music encompasses an extremely complex and extensive network of cortical and subcortical structures which integrate auditory, sensory motor and cognitive functions as well as emotional changes [2, 3]. The wide effects of music on brain function, including auditory perception, language processing, attention and memory, emotion, and motor skills have proposed the use of music as a noninvasive intervention for patients [4]. Since the 1980s, several experimental studies have been performed on fetal sensory competencies in relation to different forms of sound stimuli [5–11]. The first empirical studies were designed to explore the fetus’ hearing ability [12]. Initial responsiveness to different frequencies of sound starts from around 23 weeks of pregnancy. This is consistent with the development of neurosensory part of the auditory system which was indicated by startle response to vibroacoustic stimulation in 24 weeks of GA. Consistent responses, from all fetuses, observed between 28–30 weeks [13, 14]. The primary studies focused on the immediate response of fetus to sound stimulations and later studies followed up the effects into neonatal life. The concerning effects were memory persistence and improvements in general behavior of exposed infants. The retention of the effects of prenatal sound exposure, for instance fetal learning, suggests that fetal neurodevelopment may be positively influenced and enhanced. The repetition of stimuli shortens the time of fetal habituation, thus memory formation might happen gradually [15]. This article considers memory as a behavioral change caused by an experience, and learning as a process for acquiring memory [16]. Fetal learning has been measured using various outcome assessments including habituation testing, classical conditioning, exposure learning, heart rate (HR), and brain event-related potentials (ERPs) [17, 18]. Different forms of acoustic stimulations have been used to influence the fetus. Furthermore, there are great methodological differences in the music exposure protocols such as sound frequency and volume, means of music administration, and exposure region (directly to the maternal abdomen or in the environment). All these variables can impact the amount and quality of the sound reaching the fetus and therefore its effects [19, 20]. A previous systematic review of the effects of visual and auditory stimuli in functional fetal brain imaging also showed great variation in methodology of similar studies. This study showed that differences in the measurement strategies and study designs can lead to variable results [21].

There have been reviews concerning the therapeutic role of music during pregnancy; however, no review focused on the effects of music or speech on neurodevelopment, memory, and learning in term infants of healthy mothers. Considering the wide variation of studies and the lack of a systematic review about this concept, this study was conducted to determine the effects of sound stimulations (music and speech) on fetal memory and learning, which was assessed later in neonatal period.

## Method

### Registration and protocol

This systematic review was done in accordance with The Preferred Reporting Items for Systematic reviews and Meta-Analyses (PRISMA) [22] and registered at Pazhoohan Research Information System (registration code: 66065).

### Data sources

Four databases including the MEDLINE, Scopus, EMBASE, and Cochrane library were thoroughly searched. Also, The International Prospective Register of Systematic Reviews (PROSPERO) was searched to identify ongoing systematic reviews in the same topic. The references of recent reviews and other eligible articles were manually searched for additional studies not identified through the electronic search.

We ran our initial search strategy in May 2019 and updated it in November 2021. Controlled trials with accessible English titles, abstracts, and full-texts were included without time limitation.

### Search strategy

The search strategy was designed using the following database-specific vocabularies (MeSH, EMTREE) and additional free-text terms: (((((("Infant, Newborn"[Mesh]) OR infant) OR newborn) OR neonate)) AND (((((((((((((((((((((((("brain function") OR "brain development") OR "fetal sensory competencies ") OR "development of fetal hearing") OR "fetal development") OR "infant behavior") OR "neonatal behavior") OR "Infant Behavior"[Mesh]) OR "auditory brain development") OR "visual brain development") OR "thinking") OR "recognize ability") OR "memory") OR "neuropsycological behavior") OR "auditory attention") OR "visual attention") OR "mental function") OR "fetal learning") OR "endocrine effect") OR "metabolism effect") OR "Prenatal Exposure Delayed Effects"[Mesh])) OR ("Embryonic and Fetal Development"[Mesh] OR "Fetal Development"[Mesh])) OR "Language Development"[Mesh])) AND ((((((((((((((((("mother voice") OR "transnatal auditory learning") OR "auditory stimulation") OR "prenatal music education") OR "maternal voice") OR "maternal music") OR "antenatal training with music") OR "music effect") OR "effect of music") OR "fetal exposure to music") OR "fetal music exposure") OR "fetal exposure to mother voice") OR "fetal exposure to maternal music") OR "antenatal music education") OR "prenatal sound stimulation") OR "music during pregnancy") OR "auditory habituation").

### Inclusion and exclusion criteria

#### Types of studies

Only intervention studies (RCTs or quasi-RCTs) were considered. Publications were included without time limitation with available English titles, abstracts, and full-texts.

#### Participants

Participants comprised fetuses followed up to neonatal life and later. Non-singleton pregnancies, mothers with coexisting medical diseases, and high-risk pregnancies including diabetic patients were excluded. Studies on premature or unhealthy infants, newborns after birth, and neonates with existing congenital disease were not included.

#### Interventions

The selected studies had conducted sound stimulation including music and speech phrases.

#### Outcomes

The findings of included studies focused on the memory of the exposure, development of brain as well as improvement of neonatal behavior. The neonate’s memory of sound stimulation can be assessed by ECG, EEG, habituation tests, and behavior change. The trials that did not follow up the fetus into neonatal period were excluded.

#### Data extraction

Two reviewers screened all titles and abstracts of retrieved papers independently. Additionally, full-texts of relevant papers were screened for eligibility by two independent reviewers and the reasons for exclusion were documented for the excluded full-texts. Two researchers extracted the data separately and disagreements were discussed and resolved. The following information was extracted from the articles: the first author, publishing year, country, study design, sample size, intervention type, enrolment time, intervention duration, content of intervention, exposure region, age, state of neonate at time of test, and the type of test.

#### Quality assessment

The quality of studies was assessed according to the Joanna Briggs Institute Critical Appraisal Checklist for RCTs.

## Results

### Search results

**Fig. 1:**
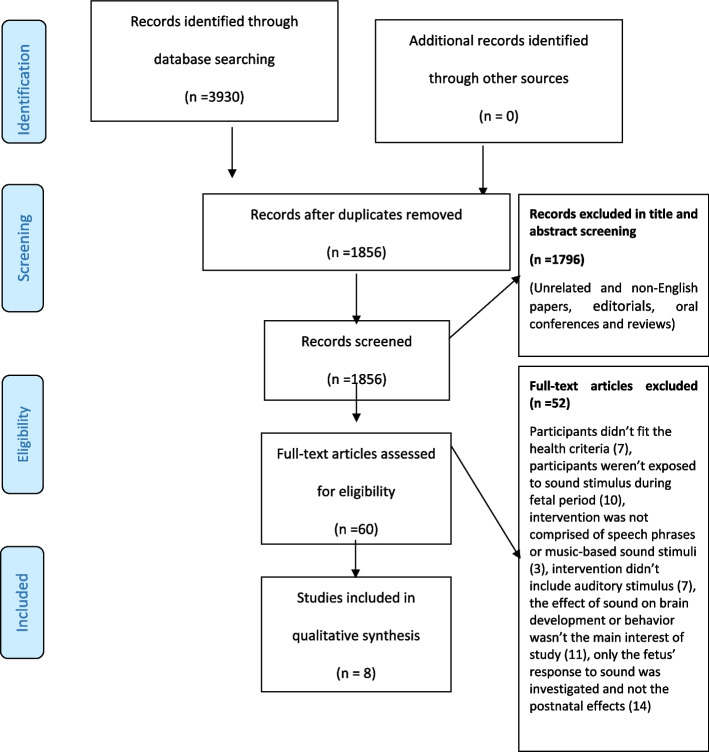
PRISMA flow diagram

Overall, 3930 titles were found in the searching process. After removal of duplicated articles using EndNote X9.3.3 literature manager software, 1856 articles remained. Initial evaluation of the titles and abstracts decreased the number of papers to 60 after elimination of unrelated and non-English papers, editorials, oral conferences, and reviews and further reduced to 8 after selecting studies which their full text met the inclusion and exclusion criteria (Fig. 1). Fifty two studies were excluded during full text assessment process, due to the following reasons: in 7 studies, participants did not fit the health criteria; in 10 studies exposure to sound stimulus was not during fetal period; in 7 studies intervention did not encompass any types of auditory stimulus; and in 3 studies the sound intervention was not comprised of speech phrases or music-based sound stimuli. Moreover, the main focus of 11 studies was not the sound’s effects on brain development and behavior. In 14 studies, only the fetus’ response to sound, and not the postnatal effect, was investigated.

### Study characteristics

The basic characteristics of the 8 included studies are summarized (Table 1). The 8 RCTs were published from 1986 to 2020. The sample size varied from 18 to 260, with a total population of 535 neonates (252 in control groups and 283 in study groups).

**Table 1:** Characteristics of the included studies

Number	Study number	Author	Country	Method											
				**Study design**	**Sample size**			**Intervention type**	**Enrolment time**	**Intervention duration**	**Content of intervention**	**Exposure region**	**Neonatal state at time of test**	**Age at time of test**	**Type of test**
					**Control group**	**Experiment group**	**Final total**								
1		Lang A et al. (2020) [23]	Austria	RCT	11	23	34, 11excluded	Rhymes with mother voice	34 GA weeks	mean:84 times of 5 min(420 min)	recorded mother and unfamiliar female voice with calm or lively rhythm	listening loudly	Preferably sleep	14.78 days (SD = 4.29) in the first recording and a mean age of 36.65 days (SD = 4.49) in the second recording	sleep-awake states based on polysomnography
2	Speech–brain coupling	Lang et al. (2020) [24]	Austria	RCT	12	22	34, 21 excluded	nursery rhymes with mother voice and non-maternal voice	< 34 GA weeks	At least 420 min	recorded mother and unfamiliar female voice with calm or lively rhythm	listening loudly	Preferably sleep	14.35 days (SD = 2.67) in the first recording and 36.48 days (SD = 3.43) in the second recording	*hd*EEG
	Heart rate analyses				13	24	37, 18 excluded								ECG
3	1	Partanen E et al.(2013) [17]	Finland	RCT	11, 1excluded	10, 2 excluded	21	music melody, speech	29 + 0 GA weeks	mean:57 times of 15 min ( 855 min)	musical melodies	listening loudly	sleep	EG:16 d CG: 13 d	ERP, MMR
	2			RCT	8, 4excluded	10, 2excluded	18	music melody, speech	29 + 0 GA weeks	mean:57 times of 15 min ( 855 min)	changed musical melodies	listening loudly		EG:144 d CG:133 d	ERP, MMR
4		Partanen E et al.(2013) [25]	Finland	RCT	16	17,11 excluded	33	nonvocal music, speech	29 + 0 GA weeks	mean: 60 times of CD (two 4-min sequences)(480 min)	three variants of pseudowords, free choice of nonvocal	listening via headphones	sleep	EG:5.5 d CG: 4 d	EEG, ERP, MMR
5		Arya R et al.(2012) [26]	India	RCT	134, 36excluded	126, 43excluded	260	music	EG:13.1 ± 2.4, CG:12.7 ± 2.9 GA weeks	173.3 (± 18.9) hours	instrumental music, natural sounds, chants from religious	listening via headphones	awake	day 2 or 3 of life	BNBAS
6	Experimental melody	Granier-Deferre C et al.(2011) [27]	France	RCT	15	13	50, 75excluded	music	35th,36th, and 37th weeks	mean: 39.24, SD = 2.05 sessions of 12 min (470 .88 min)	piano melody	listening loudly	sleep	EG:30.44 d, SD = 6.36 CG: Mean = 31.48 d, SD = 6.7	HR,HRV
	Control melody			RCT	10	12		music	35th,36th, and 37th weeks	mean: 39.24, SD = 2.05 sessions of 12 min (470 .88 min)	piano melody	listening loudly	sleep	EG:30.44 d, SD = 6.36 CG: Mean = 31.48 d, SD = 6.7	HR,HRV
7		James D et al.(2002) [28]	UK	RCT	10	10	20	music	72 h prior to elective delivery (M:38 GA weeks)	240 min	a rhythmical music (little Brown Jug)	earphone on the maternal abdomen		days 3–5	neonatal behavioral states
8	Mother voice	Decasper A et al. (1986) [29]	USA	RCT	5	9	28, 17excluded	stories with mother voice	7.5 months pregnant	67 times of story ( about 210 min)	live mother voice of stories, recorded mother voice for testing	listening loudly	awake	55.8 h postnatal	IBI of nonnutritive sucking
	Others voice			RCT	7	7		stories with mother voice	7.5 months pregnant	67 times of story ( about 210 min)	live mother voice of stories, recorded mother voice for testing	listening loudly		55.8 h postnatal	IBI of nonnutritive sucking

The participants in intervention groups underwent different kinds of sound stimulations including music (piano melodies, instrumental music, and rhythmic music) [17, 25–28] and speech phrases (speech phrases, psuedowords, stories, and rhymes) [17, 23–25, 29].

Mean enrolment time of pregnant mothers ranged from 13 to 40 weeks (gestational age). The duration of interventions was extremely different between studies, with 173.3 h of music in one study with larger population and intervention duration [26], and 210 to 855 min of music or speech in the other studies [17, 23–25, 28, 29].

Pregnant mothers and their fetuses were exposed to stimulus by listening loudly in four studies [17, 23, 24, 27, 29], via headphones in two studies [25, 26], and by devices on maternal abdomen in one studies [28].

The infant’s age at the time of test was different across studies; neonates were tested at first week of life in four studies [25, 26, 28, 29], and at the end of first month and through fifth months of life in two studies [17, 27]. Two studies evaluated the memory of infants two times, during second and fifth weeks of life [23, 24]. Five studies examined the infants during sleep periods [17, 23–25, 27] and two studies tested them while they were awake [26, 29].

The type of test used to measure outcomes was EEG in three studies [17, 24, 25], ECG and HR in two studies [24, 27], neonatal behavioral assessment in two studies [26, 28], habituation test in one study [29], and sleep-awake states based on polysomnography in one study [23].

The eight included studies reported different, and in some cases controversial, results of the sound stimulation on two comparing groups.

### Findings

The specific findings of the included studies are summarized in Table 2. Partanen et al. has reported long-term plastic effects, lasting for several months, on the developing brain in addition to boosted neural responsiveness to the music used in the fetal training [17]. Similarly, in another study by Partanen et al., the results indicated modulation of neural responsiveness and enhancement of neural commitment in neonates that were exposed to selected speech stimuli during the prenatal period [25]. In the study conducted by Arya et al., the results of behavior assessment in tested neonates demonstrated that prenatal music exposure to mother had significant favorable effects on neonatal behavior [25]. Other studies has referenced the positive effects of prenatal sound intervention in forms of habituation, learning and memory formation. Decasper et al. found that the neonates had learned and remembered some features of the acoustic cues that helped them prefer their mother voice to others’ [29]. Also, in a randomized study by James et al., examination of neonatal behavioral states, suggested that a simple form of fetal programming or learning has happened for prenatally stimulated fetuses [28]. Moreover, Granier et al. showed that repetitive prenatal exposure to a specific melody impacted neonate’s auditory perception and formed a memory of the sound stream with a retention interval from 3–4 days to six weeks [27].

**Table 2:** Specific findings

Number	Author	Specific finding	Effect size
**1**	Lang A et al. (2020) [23]	Effect of Auditory Stimulation on Sleep–Wake-States	The time spent in the three behavioral states: *F*(1.53, 49.03) = 14.71, *p* < 0.001
		Effect of Voice Familiarity on Sleep–Wake-States	STATE*VOICE: *F*(1.58, 51.30) = 1.71, *p* = 0.196
		Effect of Rhyme Familiarity on Sleep–Wake-States	STATE*RHYME: *F*(1.44, 31.70) = 0.21, *p* = 0.752
		Effect of Auditory Stimulation on Physiology (HR)	GROUP (*F*(1, 29) = 8.99, *p* = 0.006
**2**	Lang et al. (2020) [24]	Effect of Rhyme Familiarity on Infant’s Heart Rate	OR; *F*(1, 21) = 0.01, *p* = 0.972,
		Effect of Voice Familiarity on Infant’s Heart Rate	VOICE *F*(1, 32) = 0.10, *p* = 0.750
		Effect of Rhyme Familiarity on Infant’s Brain Physiology	RHYME _ FREQ (*F*(2, 46) = 6.16, *p* = 0.004, after correcting for multiple comparisons:*p* > 0.0166
		Effect of Voice Familiarity on Infant’s Brain Physiology	VOICE (*F*(1, 36) = 9.39, *p* = 0.004
**3**	Partanen E et al.(2013) [17]	MMRs for vowel identity changes of the syllable	Learning group: t(16) = 2.536, *P* < 0.022, control group: t(15) = 2.577,*P* < 0.021
		MMRs for pitch changes of the syllable	Learning group: t(16) = 3.640, *P* < 0.002 Control group: t(31) = 2.122, *P* < 0.042, d = 0.763
**4**	Partanen E et al.(2013) [25]	responses to the unchanged sounds	Learning group at birth: (t(19) = 2.11, *p*,0.049, d = 0.97) Learning group at the age of four months (t(16) = 3.33, *p*,0.004, d = 1.68)
		Amplitudes of response at birth	unchanged (*r* = 0.74, *p*,0.015, R2 = 0.54) and changed sounds (*r* = 0.68, *p*,0.032, R2 = 0.46)
**5**	Arya R et al.(2012) [26]	BNBAS	Habituation (ES 1.05, 95% CI 0.53–1.57, *P* = 0*.*0001), orientation (ES 1.13, 95% CI 0.82–1.44, *P* < 0*.*0001), motor performance (ES 0.25, 95% CI 0.0–0.5, *P* = 0*.*0479), range of State (ES 0.31 95% CI 0.17- 0.45 *P* < 0.0001), regulation of state (ES 0.54 95% CI 0.28, 0.80 *P* < 0.0001), and autonomic stability (ES 0.26 95% CI 0.06, 0.46 *P* = 0.0102)
**6**	Granier-Deferre C et al.(2011) [27]	Heart Rate analysis of the subjects with a cardiac deceleration	mixed ANOVA on the z-scores: Group, *F* (1, 28) = .30, *p* = .59, Melody, *F* (1,28) = 1.35, *p* = .26, Group x Melody interaction, *F* (1, 28) = 1.22, *p* = .28 Time, *F* (149, 4172) = 13.02, *p*,.00001, Time x Group, *F* (149, 4172) = 1.38, *p*,.0017, Time x Melody, *F* (149, 4172) = 1.79, *p*,.00001,
**7**	James D et al.(2002) [28]	Neonatal behavioral states	median time to a state change (*P* = 0.01) median time to S1 (*P* = 0.06) median time to exhibited awake states (S4 and S5) (*P* = 0.05) transitions during exposure to music compared to the baseline period (*P* = 0.05 and *P* = 0.04)
**8**	Decasper A et al. (1986) [29]	IBI of nonnutritive sucking	baseline conditional probabilities of target and novel stories: t(11) < 0.1 reinforcement ratios of matched-subject pairs target-story ratios: t(11) = 2.68, *p* < .05, novel-story ratios: t(11) < 0.1

*RCT* Randomized Control Trial, *EG* Experiment Group, *CG* Control Group, *GA* Gestational Age, *EEG* Electroencephalography, *hdEEG* high-density Electroencephalography, *ECG* Electrocardiography, *MMR* Mismatch Response, *ERP* Event-related Potentials, *BNBAS* Neonatal Behavioral Assessment, *HR* Heart Rate, *HRV* Heart Rate Variability, *IBI* Interburst intervals, *ES* Effect size

* interaction

In contrast, Lang et al. found no stimulus-specific effect of sound, rhyme or voice familiarity on the newborns’ behavioral states in the prenatally exposed group. Nevertheless, a general calming effect of the experienced stimulus was found, which indicated fetal learning [23]. Another clinical trial by Lang et al. showed that newborns react distinctly to the maternal voice at second and fifth weeks of birth, identifiable with ECG and EEG. Therefore, it appears that basic memory traces are formed in fetal period and effect the neonate’s autonomic and neuronal reactions to sound stimuli [24].

### Quality assessment of studies

The risk of bias assessment of included studies is summarized (Table 3). All studies had a good general quality based on JBI critical appraisal checklist for RCTs. Four trials had a high risk of selection bias [17, 25, 27, 29]; in all studies, treatment groups were similar at the baseline characteristics. Blinding of participants and those delivering the treatment was not applicable in the studies since mothers listened to the music directly or they could be aware of vibrations produced by the device (earphones, artificial larynx) on their abdomen. In five trials, blinding of outcome assessor was not reported, so there was a high risk of detection bias [17, 23–25, 27]. In one study, the reasons of loss to follow up were not discussed, thus there was a risk of attrition bias [25]. Low risk of information bias was detected for all studies since outcomes were measured in a reliable and the same way for the compared groups. Participants were analyzed in the groups to which they were randomized in all trials. Appropriate statistical analysis was used in all studies. The designs of all trials were appropriate for the topic.

**Table 3:**
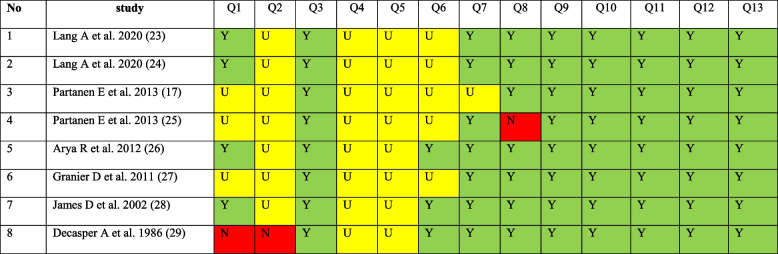
Quality assessment of the included studies [17, 23–29]

(*Y* Yes, *N* No, *U* Unclear; JBI critical appraisal checklist for randomized controlled trials: Q1 Was true randomization used for assignment of participants to treatment groups?; Q2 = Was allocation to treatment groups concealed?; Q3 = Were treatment groups similar at baseline?; Q4 = Were participants blind to treatment assignment?; Q5 = Were those delivering treatment blind to treatment assignment?; Q6 = Were outcome assessors blind to treatment assignment?; Q7 = Were treatment groups treated identically other than the intervention of interest?; Q8 = Was follow-up complete, and if not, were strategies to address incomplete follow-up utilized?; Q9 = Were participants analyzed in the groups to which they were randomized?; Q10 = Were outcomes measured in the same way for treatment groups?; Q11 = Were outcomes measured in a reliable way?; Q12 = Was appropriate statistical analysis used?; Q13 = Was the trial design appropriate, and any deviations from the standard RCT design (individual randomization, parallel groups) accounted for in the conduct and analysis of the trial?)

### Level of recommendations

The JBI Recommendation Leveling Checklist was used to evaluate the results of included Studies, the results of which are presented in the Table 4.

**Table 4:** Grading of evidence based on JBI grading

Number	Author	Recommendations	Grade
1	Lang A et al. (2020) [23]	Prenatal experience or “fetal programming” may have an effect on behavioral (sleep/wake states) and physiological (heartrate) reactions just weeks after birth as evident in less waking periods and HR habituation to stimuli heard already in the last trimester before birth. Parents and societies should be aware of such effects and may consider this in their parenting methods even before birth. This is not to say that we are in favor of overambitious stimulation of the fetus in order to maximize learning even before birth. Rather we call into attention that much of what a fetus is exposed to before birth—whether it is parental movements, touch, music, or speech—may shape the infants’ physiology and later perception of the world	A
2	Lang et al. (2020) [24]	Our results indicate that newborns show distinct reactions to the maternal voice already at birth (two and five weeks) even on a physiological level and identifiable with ECG and EEG. Furthermore, it appears that basic memory traces are formed in utero and shape the newborn’s autonomic and neuronal reactions to speech and voice stimuli, namely, in such a way that newborns familiarized to nursery rhymes prenatally show distinctly different reactions than newborns being naïve in this respect. This again emphasizes the importance of the prenatal environment and calls into attention that already at these times the brain is tuned or “programmed” for the postnatal environment predicted and most likely experienced	A
3	Partanen E et al.(2013) [17]	These results indicate that auditory experiences during the fetal period can induce changes in neural processing and therefore have several important practical implications. First, these results indicate that the shaping of the central auditory system begins before birth. Repeated exposure to certain types of sounds leads to the development of neural memory traces for these sounds, as suggested by the strengthening of the activation in the MMR time range to changes in the learned material in the learning group. Thus, it appears likely that hearing a great deal of speech before birth may have positive effects, preparing the neural apparatus for the accurate analysis and discrimination of the fine acoustic features of speech. These early experiences may, then, affect the individual’s later abilities of speech perception and language acquisition	B
4	Partanen E et al.(2013) [25]	Our results show that prenatal exposure to music can have long-term plastic effects on the developing brain and enhance neural responsiveness to the sounds used in the prenatal training. Furthermore, we found that these plastic changes are long lasting. These findings have several practical implications. First, since the prenatal auditory environment modulates the neural responsiveness of fetuses, it seems plausible that the adverse prenatal sound environment may also have long-lasting detrimental effects. Such environments may be, for example, noisy workplaces and, in case of preterm infants, neonatal intensive care units. Furthermore, as prenatal exposure still affected the ERP responses months after birth, additional fetal exposure to structured sound environments might be beneficial for supporting the auditory processing of, for example, infants at risk for dyslexia in whom basic auditory processing was shown to be impaired	B
5	Arya R et al.(2012) [26]	In conclusion, this study provides preliminary evidence that maternal music exposure beneficially affects neonatal behaviour. A trained clinician can utilize the behavioural organization of the newborn infant to gain insights into the intrauterine experience and the perinatal events which may have influenced the neonate’s CNS organization	A
6	Granier-Deferre C et al.(2011) [27]	3-weeks of prenatal exposure to a specific melodic contour affects infants ‘auditory processing’ or perception, i.e., impacts the autonomic nervous system at least six weeks later, when infants are 1-month old. The long-term memory for the descending melody is interpreted in terms of enduring neurophysiological tuning and its significance for the developmental psychobiology of attention and perception, including early speech perception, is discussed	B
7	James D et al.(2002) [28]	The first prospective randomized controlled study to demonstrate that fetal exposure to a complex sound stimulus results in the development of altered behavior in the fetus and the occurrence of altered behavior in the newborn period compared to unexposed controls. We have not examined whether this effect is specific to this stimulus or sound exposure in general. Furthermore, there is no information that such effects are either long lasting or beneficial	A
8	Decasper A et al. (1986) [29]	The study provides the first direct evidence that prenatal auditory experience with a particular maternally generated speech stimulus influences the reinforcing value of that stimulus after birth. The present study suggests noninvasive, ethically acceptable methods to further study the effects of prenatal auditory stimulation on postnatal auditory function and development, especially the development of speech perception	B

## Discussion

Overall seven studies showed that the tested neonates had learned or remembered the prenatal sound stimulus [17, 23–25, 27–29] and one study showed that the prenatally stimulated infants performed significantly better on orientation and habituation in a neonatal behavior test [26]. These findings suggest that stimulus-specific memory traces are formed during fetal period and shape the neonate’s autonomic and neuronal reactions to sound stimuli. Moreover, music exposure during pregnancy might have beneficial influence on neonatal behavior responses, which is an indicator of the integrity of nervous system at several levels. However, our findings also imply that because the fetal neural system is malleable to the surrounding sounds, it is also vulnerable to potential harmful environmental acoustic stimuli. Nonstandard, unstructured, and unusual acoustic stimulation, which the fetus could perceive as noise, cannot be recommended until further researches on such stimulation have been thoroughly conducted [30].

There have been multiple clinical trials regarding the impact of prenatal sound-based interventions on different aspects of fetal development. Systematic reviews and meta-analyses has been conducted to evaluate the effects of sound stimulations during pregnancy. Two of these studies indicated that music therapy as a non-pharmacological approach, can reduce anxiety levels during pregnancy and labor [31, 32]. Music interventions may decrease stress levels and physiological anxiety related indexes such as HR, systolic and diastolic pressures. Two other systematic reviews showed positive influence of music therapy on well-being and quality of life after birth in premature neonates in the neonatal intensive care unit (NICU). Music therapy can significantly improve preterm infant's HR, respiratory rate, anxiety level, and oral feeding volume [33, 34]. A systematic review by Carvalho et al. explored the immediate responses of the fetus to mother voice [35]. But this review did not investigate the memory of neonates of mother voice experienced in fetal period. Another study conducted by He et al. systematically reviewed the effect of prenatal music therapy on fetal and neonatal status; however, the neonatal effects weren’t discussed properly [36]. Moreover, studies with unhealthy participants such as pre-eclamptic and diabetic mothers, and pregnant women during labor were included. This study didn’t investigate fetal learning and the memory formation of prenatal music exposure. Therefore, this is the first systematic review to determine the effect of sound stimulations including music and speech, on fetal learning, memory and neonatal behavior, which was assessed later in neonatal period. This article could provide useful information for family-centered maternity care, pregnant women and neurodevelopment researchers.

Of the eight studies included, all had good general quality based on having more low-risk domains than high-risk ones. In four studies, application of randomization had a high risk of bias [17, 25, 27, 29]. There was high risk of bias in terms of allocation concealment in all of the studies. In five studies, the blinding of outcome assessor was not reported [17, 23–25, 27]. There was a high risk of bias concerning the reasons of loss to follow up in one study [25]. The final results might have been influenced by the high risk of selection and detection bias. Hence, there is a need for rigorously designed RCTs to provide reliable information on this concept.

There was considerable variability in the existing literature. Dissimilarities were found in the intervention process and outcome measurement. The sample size of trials varied from 18 to 260. The fetus’ age in the start of exposure ranged from before 20 weeks of gestation to 72 h prior to elective delivery. Considering the fact that the onset of fetal hearing occurs at about 23 weeks of gestation and completes at 31 weeks, sound exposure in most of the studies took place at third trimester of pregnancy [13]. However, it is not clearly known when the beneficial effect of fetal sound stimulation starts and thus optimal timing for such intervention in clinical practice cannot be determined. There is a lack of evidence concerning the role of timing of prenatal sound simulation [26].

The types of sound stimulation applied in the studies could be divided into music and speech by maternal or non-maternal voice. There have been other studies evaluating the effects of VAS on fetal learning and habituation [37]. Although, it’s important to note that using VAS is not recommended due to the potential adverse effects on fetal auditory system and the lack of reliable evidence on this matter [38].

Studies have used recorded as well as live maternal speech to stimulate the fetus, though the recorded music was not often described in details. In a research performed by Krueger et al. the fetal response to maternal live voicing was compared to a recorded format. Although the outcomes were difficult to interpret, the different response was easily detected [39]. All studies attempted to use novel stimuli with no possibility of exposure before and after birth, other than the controlled time. There were different ways of comparison between types of sound stimulation among included articles. The content of stimulation was altered in postnatal testing in the study by Decasper et al. in order to investigate the specificity of the formed memory [29]. Partanen et al. changed a number of notes in the original melody and showed that the responses to the unchanged music were greater in the study than control group, both at birth and at the age of four months [17]. Granier et al. used two ascending and descending piano melodies which had inverse contours, but similar spectra, same duration, tempo, rhythm, and amplitude. The results showed significant change in HR to the familiar melody compared to unfamiliar one in the study group [27]. Two studies by Lang et al. compared the effect of rhythm and familiarity of the voice of nursery rhymes in newborns’ response. Interestingly, the results revealed that familiarity of rhyme and voice (mother vs. other female) had no significant difference on infant’s HR and sleep-awake state. Though, the brain-level data demonstrated a distinct response of the neonate’s brain to the familiarity of voice [23, 24].

Standardization of the intensity of sound stimulation appeared to be an important challenge in the studies. Most of the studies used sounds with more than 80 dB of intensity, since diminishing of about 20 dB is expected through maternal abdomen [40]. Also, the maternal voice undergoes no or little attenuation when transmitted to the uterus [41–44]. Sound levels between 80 and 110 dB adequate to reach the fetal cochlea [13]. It is important to control the sound volume, due to the fact that intensity of sound influences the habituation time for fetus [45]. In the study conducted by Arya et al., mothers were allowed to decide about the volume of music, since the stimulus was not directly applied to maternal abdomen [26].

Frequency is another characteristic of acoustic stimulations that can impact the quantity and quality of the sound that reaches the fetus, and consequently its effects. Fetal detection of frequency changes happens in womb early in fetal development, at 28 weeks of pregnancy [46, 47]. Fetuses first respond to low frequency 250 or 500 Hz tones at about 25–27 weeks of gestational age, and afterwards to the 1000 or 3000 Hz tones by 29–31 weeks of gestational age [13]. In two studies, the range of frequency used for stimulation was about 100–1000 Hz [25, 27]. The frequency of sound was not reported in the other six studies. Further studies are required to determine the optimal frequency of music to affect the fetus in utero. Studies should provide clear reporting of the sound frequency and consider the possible adverse effects of uncontrolled sound stimuli. Future researches are recommended to avoid prolonged fetal exposure to low-frequency sound levels (< 250 Hz) higher than 65 dB [48, 49].

The duration of intervention was decided based on each study’s objectives. It ranged from 210 to 855 min for studies designed to investigate learning and memory [17, 23–25, 27–29], and about 173 h for a study designed to investigate changes in neonatal behavior [26]. Partanen et al. reported that longer exposure to speech material, such as psuedowords, may generally improve speech discrimination [25]. Nevertheless, none of the included articles focused on the influence of stimulation duration on the outcomes. Another study conducted on premature infants examined the effect of sound intervention until the infants reached term using cranial ultrasonography. The results showed that the degree of the right and left auditory cortex development was not significantly correlated with the duration of music exposure [50].

The age of neonates at time of test varied between studies; four studies tested the neonates at first week of life so as to evaluate the hypothesis of learning during prenatal period and transferring the memory into neonatal life [25, 26, 28, 29]. The other studies tested the infants at 2 and 5 weeks of life, the end of first month, and through 5 months of age in order to assess memory retention [17, 23, 24, 27]. No studies were found to investigate the retention of memory in the exposed infants after five months of age.

Most of the studies tested the infants during sleep periods, which might diminish the observable effects of the stimulation [17, 23–25, 27]. Although, recognition of familiar stimuli can occur during sleep in adult brain [51].

There were different kinds of outcome assessment methods within studies as follows:

- 1- EEG: in 3 studies EEG, mismatch response (MMR), and ERPs were used for detecting memory traces of sounds experienced in the womb [17, 24, 25].
- 2- ECG and HR: in 2 studies the cardiac response elicited by prenatally exposed melodies was used for testing the neonates [24, 27].
- 3- sleep-awake states based on polysomnography: in one study changes in behavioral states (quiet sleep, active sleep, transitional sleep, awake) to auditory stimulation was examined with polysomnography. This included monitoring infants with electromyogram, electrooculogram, ECG and videography [23].
- 4- Neonatal Behavioral Assessment: in one study Brazelton Neonatal Behavioral Assessment Scale (BNBAS) was employed for outcome evaluation [52]. One other study used conventional neonatal behavioral analysis criteria [53]. These tests examine the integrity of neonatal nervous system at different levels.
- 5- Nonnutritive sucking: alteration of sucking behavior in newborns when exposed to a familiar and unfamiliar recorded voice was utilized as a measurement tool in one study [29].

The region of exposure was an indicator factor of the underlying mechanism of the resulted effects. Stimulation was either direct (using devices on the maternal abdomen) [28] or indirect (mothers listened to music with headphones) [25, 26]. It’s important to note that earphones or other sound producing devices are not recommended to be used directly attached to the pregnant woman's abdomen [48, 49]. There was a better practical implication when the mother used conventional headphones over her ears due to its easier adaptability to the routine lifestyle [26]. In the studies which the sound stimulation was transmitted via environment (listening loudly to sound, reading stories out loud by mother), both direct and indirect mechanisms could be involved [17, 23, 24, 27, 29].

There were two probable mechanisms for the effects of prenatal music exposure on neonatal behavior, one of which was the indirect mechanism. The indirect effects are likely to be mediated by endocrine changes in mother [26]. Music is known to have several endocrine effects including increased growth hormone, which modifies the production of certain cytokines, increased levels of ovarian steroid secretion, alterations in the biorhythms, cortisol, testosterone, and estrogen levels [43, 44]. Corticosteroids [1] regulate growth of neuroblasts, myelination, and metabolism in developing brain in different ways [33]. Thus, the indirect mechanism was likely to be mediated via endocrine changes of stress reduction in mothers that caused enhancement of fetal neural network [25].

The direct mechanism included neural mechanisms, like music processing. Music perception along with auditory signal transduction triggers a sequence of motor, cognitive, and emotional processes that evokes a number of brain areas, unilaterally and bilaterally [46]. Another direct mechanism was neuro-physiological adaptive process. This process is mediated by the autonomic nervous system and aims at tuning the auditory system. The neuro-physiological adaptive mechanism might form a better neuro-functional organization of fetal auditory system by structuring cellular and synaptic plasticity and improving receptive field selectivity [27]. As a result, fetal sound stimulation might develop a more effective neural network for detection of the changes in sounds. However, further researches are needed to shed light on the precise mechanisms of the discussed effects.

### Limitation

The main limitation of this systematic review was the heterogeneity of intervention and outcome assessment methods across the studies that made it difficult to reach a consistent conclusion. The search strategy and study selection processes were restricted to English-language publications and limited databases. Furthermore, it should be noted that most of the studies had high risk of selection and detection bias that might influence the final conclusion.

### Suggestion and implication

There is a need for methodologically strong RCTs about this concept with rigorously designed interventions, consistent outcomes, and standardized reporting measures. Future studies may include comparisons of different intervention durations and sound stimulation types. Specific effects of different types of music including instrumental music compared to vocal one and maternal voice versus other voices should be considered in future studies. Recommended areas for further research are the mechanisms of the effect of music on fetus, the association between behavioral development of newborn and intrauterine central nervous system (CNS) organization, and more long-term follow-up studies. Using music during pregnancy may have positive implications for maternal-neonatal bonding after birth [35]. This field of study could be useful for family-centered maternity care to provide a novel and pleasant care for pregnant mothers and neuro-researchers to extend their measures for understanding neurodevelopment process.

The findings of this study emphasizes the importance of the prenatal environment on fetal development, therefore, the influence of adverse prenatal sound environment on fetus is an essential field to be studied in future researches. Studies should follow safety recommendations strictly and provide clearer reporting on sound stimulation characteristics [48, 49]. It’s important to note that although this review shows evidence that the fetus can learn, it does not mean that they need to be taught anything. Misinterpretation of the data has resulted in the development of commercial products that promote use of headphones applied to the pregnant abdomen that play certain types of music and sounds to help "improve" prenatal brain development. This might be a potentially harmful practice due to the currently unsufficient knowledge on the matter.

## Conclusion

The findings of this study suggest that sound stimulation (music and speech) can form stimulus-specific memory traces during fetal period and shape the neonatal autonomic and neuronal reactions to sound stimuli. Also, fetal music exposure might have beneficial effects on neonatal behavior responses, which is an indicator of the integrity of nervous system. However, these outcomes also imply that the fetal neural system is vulnerable to potential harmful surrounding sounds. There is a need for methodologically strong RCTs that follow safety recommendations strictly and provide clearer reporting on sound stimulation characteristics. Mechanisms of the effect of music on fetus, the influence of adverse prenatal sound environment on fetus, the association between behavioral development of newborn and intrauterine CNS organization, and more long-term follow-up studies are the recommended areas for further research.

## Data Availability

The datasets used and/or analyzed during the current study available from the corresponding author on reasonable request.

## Abbreviations

VAS: Vibroacoustic stimulation
ECG: Electrocardiography
EEG: Electroencephalography
HR: Heart rate
ERPs: Brain event-related potentials
PRISMA: The Preferred Reporting Items for Systematic reviews and Meta-Analyses
NICU: Neonatal intensive care unit
MMR: Mismatch response
BNBAS: Brazelton Neonatal Behavioral Assessment Scale
CNS: Central nervous system
